# A Multi-Functional View of Moral Disengagement: Exploring the Effects of Learning the Consequences

**DOI:** 10.3389/fpsyg.2017.02286

**Published:** 2018-01-26

**Authors:** C. Justice Tillman, Katerina Gonzalez, Marilyn V. Whitman, Wayne S. Crawford, Anthony C. Hood

**Affiliations:** ^1^Narendra P. Loomba Department of Management, Baruch College (CUNY), New York, NY, United States; ^2^Management Department, University of Alabama, Tuscaloosa, AL, United States; ^3^Management Department, University of Texas at Arlington, Arlington, TX, United States; ^4^Management, Information Systems and Quantitative Methods Department, University of Alabama at Birmingham, Birmingham, AL, United States

**Keywords:** moral disengagement, conservation of resources (COR), unethical behavior, guilt, shame

## Abstract

This paper takes us beyond the unethical act and explores the use of moral disengagement as a multi-stage, multi-functional regulatory, and coping mechanism that not only allows individuals to engage in unethical behavior, but also manage the negative emotions (i.e., guilt and shame) from learning the consequences of such behavior. A resource-based lens is applied to the moral disengagement process, suggesting that individuals not only morally disengage prior to committing an unethical act in order to conserve their own resources, but also morally disengage as a coping mechanism to reduce emotional duress upon learning of the consequences of their actions, which we describe as *post-moral disengagement*. These assertions are tested using a scenario-based laboratory study consisting of 182 respondents. Findings indicate that individuals will morally disengage in order to commit an unethical act, will experience negative emotions from having learned of the consequences, and then will engage in post-moral disengagement as a coping mechanism. In addition, the findings suggest that guilt and shame relate differently to moral disengagement.

## Introduction

By and large, most moral individuals have an innate desire to do what is right (Gentile, 2010). The process by which individuals determine what is right, however, is an increasingly complex one (Treviño et al., 2014). Ethical decision-making, in its most basic form, involves a series of cost-benefit analyses, such that when faced with a moral dilemma, individuals are motivated to compare the costs of violating moral standards with the perceived benefits of adhering to such standards. Yet, often times the costs associated with the decision's negative return for perpetrators and those affected by their actions are not fully considered. Given that all actions generate consequences, whether they are large or small, positive or negative, individuals are likely to experience additional emotional discomfort after learning about the consequences resulting from their unethical act. Although extant research has explored the emotions present at the decision-making time, the anticipated emotional responses of engaging in an unethical act, and the emotional outcomes of having violated one's moral standards (Higgins, 1987; Gaudine and Thorne, 2001; Lowenstein et al., 2001; Morris et al., 2002; Sayegh et al., 2004; Tenbrunsel and Smith-Crowe, 2008), little is known about the emotional reactions produced as a result of *learning about the consequences* of one's unethical behavior (Tillman et al., 2017). Hence, the present study aims to address this gap by examining the process by which individuals, who view themselves as moral (i.e., moral judgment) elect to engage in unethical acts (i.e., moral actions) and deal with the consequences of their acts (Blasi, 1980, 1983; Tillman et al., 2017).

Moral disengagement theory (MDT) suggests that individuals tend to cognitively separate the moral component from an otherwise unprincipled act in order to rationalize engaging in it (Bandura et al., 1996; Bandura, 1999, 2015). That is, individuals employ one or more mechanisms to disengage self-sanctions from unethical behavior in order to validate and rationalize the behavior, allowing them to engage in or live with the consequences of their unethical acts. Yet, MDT does not generalize beyond the unethical act. In light of research that suggests that thoughts of future unethical events evoke stronger emotional reactions than thoughts of previous unethical events (Van Boven and Ashworth, 2007; Caruso, 2010), we deem it necessary to further enhance our understanding of the moral disengagement process. By exploring the emotional responses that result from learning the consequences of one's actions and examining the mechanisms used to cope with these emotions we demonstrate the ongoing cycle of moral disengagement; that is, individuals do not necessarily stop morally disengaging after having talked themselves into engaging in the unethical act. The process of morally disengaging is a multi-stage, multi-functional regulatory, and coping mechanism that not only allows individuals to engage in unethical behavior, but also manage the negative emotions that may arise from learning the consequences of such behavior.

Using a resource-based view of ethical decision making as a theoretical framework, we examine simulated interactions designed to illustrate the complex interplay between the moral dilemmas that often emerge in the context of social relationships. This framework is rooted in conservation of resources theory (COR; Hobfoll, 2001; Halbesleben et al., 2009). COR may help to explain the conditions through which individuals are motivated to direct their existing resources to protect, replenish, or build resources (Hobfoll, 2001; Halbesleben et al., 2009). According to this model, resources may include emotional energy, social relationships, time, and attention (Hobfoll, 1989). When resources are threatened by factors such as excessive obligations or relationship demands, individuals are motivated to take actions to protect their available resource stocks from undue depletion. Perceptions of threat may provoke a disinvestment of resources such as through avoidance, denial, or withholding effort. Conservation of resources also may trigger an investment of resources such as when an offender attempts to avoid the loss of a relationship by apologizing to an offended party in the wake of inappropriate behavior.

Our study contributes to the literature in a number of ways. First, we supplement existing research on moral disengagement by advancing a resource-based view of ethical decision-making. We view unethical decision-making as a disinvestment of resources in others in order to preserve, recover, or maximize one's own resources (Nelissen et al., 2013). Drawing on COR theory, we offer a novel reinterpretation of moral disengagement that highlights efforts taken to cope with the emotional strain associated with moral dilemmas and unethical decision outcomes. Specifically, we describe how moral dilemmas, particularly those involving people in close personal relationships, place competing demands on decision makers' resources. Personal relationships such as those among friends or workplace colleagues, involve mutual expectations of reciprocal investment, continual maintenance, and avoidance of harm (e.g., Hansen, 1999; Methot et al., 2016). However, legitimate efforts to maximize or protect one's own resources may be at odds with the resource expectations associated with upholding the standards of friendship; self-preservation may inadvertently cause harm to a friend or to a friendship. Navigating these competing demands of upholding moral standards on the one hand and honoring the obligations of friendship on the other, may drive difficult resource allocation decisions. Some choices conserve one's own resources while simultaneously deprive friends of others. Such dissonance is likely to trigger shame and guilt, particularly when decisions cause a potential or actual loss to self or others.

Next, we argue that ethical decision-making and moral conduct research has focused largely on what happens prior to engaging in the unethical act and the immediate emotional outcomes that result from having violated one's moral standard (e.g., Ferrell and Gresham, 1985; Higgins, 1987, 1989; Jones, 1991; Robertson and Ross, 1995; Bandura et al., 1996; Bandura, 1999, 2002; Gaudine and Thorne, 2001). Missing is theory that addresses how individuals “live with themselves” after learning about the consequences of their actions and seek to salvage the relationship with individuals who may have been negatively impacted. Viewing moral disengagement via a COR lens, we suspect that to ease or eliminate the emotional burden associated with moral violations and the consequences of such, individuals may engage in *post-moral disengagement*, or moral disengagement after the unethical act. In addition, engaging in post-moral disengagement in the context of personal relationships, such as friendship, provides a mechanism for individuals to validate or rationalize their unethical act to the persons negatively affected by the behavior, creating an avenue for protecting the relationship. Thus, we suggest that the process outlined in this COR view of moral disengagement is employed by individuals to both disengage self-sanctions before engaging in the unethical act (thereby cognitively discounting the potential costs) and after the act as a means of reducing or reversing the emotional burdens that escalate as the magnitude of the consequences of one's actions are realized.

## Theoretical framework

### Conservation of resources theory

Conservation of resources theory (COR) highlights individuals' motivation to defend, recover and build resources (for a recent review, see Halbesleben et al., 2014). Perceived threats encourage actions to protect resources from loss. In the absence of recognized threats, individuals are motivated to invest their existing resources to amass more resources. In the wake of an actual loss, individuals are motivated to invest their available resources to interrupt downward spirals of loss, replenish that which was lost, and to build resource reserves to address future threats (Halbesleben and Bowler, 2007). Moreover, research in this area suggests that individuals may experience emotional exhaustion and burnout if they perceive an inadequate return from prior resource investments (Halbesleben and Bowler, 2007).

An emerging perspective in the literature involves resource conservation efforts among those in close personal relationships, such as friendships (e.g., Hood et al., 2016; Methot et al., 2016). The relationship among friends serves as a conduit through which resources such as feelings of identity, inclusion and social and emotional support (Umphress et al., 2003) flow from one to the other. Friendships are characterized by reciprocal exchange of resources, frequent communication, trust, and emotional intensity (Granovetter, 1973). Despite these benefits, friendships may have a dark side. Maintaining effective friendships comes with a number of expectations that may lock friends into unwanted patterns of exchange that place an undesirable drain on friends' personal resources (Hansen, 1999; Methot et al., 2016).

Continual resource investments are required in order to develop and maintain friendships (Hansen, 1999; Methot et al., 2016). Moreover, friends are expected to avoid actions that might threaten the relationship and impede the interpersonal flow of social and emotional resources. Friends may be resentful of unreasonable demands placed on their limited resources which may trigger a perceived threat. For the sake of self-preservation, individuals may delay or deny interpersonal resource investments (e.g., honoring a favor from a friend). However, such actions may be perceived as violations of friendship obligations and cause social and emotional distress for all involved parties. When faced with a moral dilemma in which an individual is forced to evaluate and compare the costs of violating one's moral standards (as opposed to violating norms of friendship), decision makers may display a tendency to engage in moral disengagement.

### Moral disengagement theory

**Figure 1:**
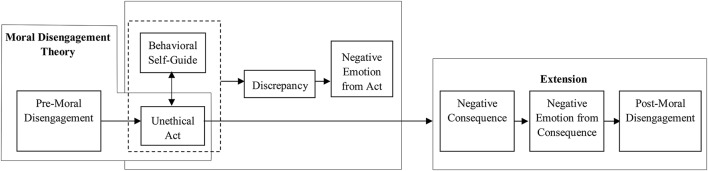
A process model of post-moral disengagement.

Depicted in the first two boxes in Figure 1, moral disengagement details the process by which individuals rationalize engaging in immoral behavior (Bandura et al., 1996; Bandura, 1999, 2002). To avoid feelings of self-condemnation or loss, individuals are motivated to refrain from engaging in behaviors that violate their moral standards. When tempted to behave unethically, individuals will seek to validate or rationalize the decision by disengaging self-sanctions from the behavior.

Moral disengagement posits that there are four major sets of disengagement practices that individuals rely on to justify unethical behavior (Bandura, 1999). The first set of practices involves reconstruing the conduct so that it is not considered immoral (Bandura, 2002). For example, a healthy employee, calling in sick from work can be cognitively reframed as self-care. Another means of reconstruing one's conduct involves the use of euphemistic labeling to sanitize unprincipled acts or pass the blame (e.g., referring to patients by their room number enables depersonalization). An individual also may resort to advantageous comparison in which one's unethical behavior is compared with an equally or greater unethical behavior for the purposes of exoneration. The second set of disengagement practices consists of minimizing personal involvement. An individual may displace his or her responsibility by minimizing or obscuring the role that he or she plays in the unethical act. Personal involvement also may be minimized by diffusing one's responsibility. As Bandura (2002) notes “when everyone is responsible, no one really feels responsible” (p. 107). Spreading the blame serves to obscure personal agency and weaken the exercise of moral control. The third disengagement practice involves misrepresenting or discounting the resulting negative consequences. Individuals may be more likely to engage in an unethical act when they do not have to face the recipient of the mistreatment. The fourth set of practices consists of blaming or devaluating the recipients of the mistreatment. Assigning the blame to someone or something else makes one's unethical actions excusable and may even make the individual who engages in the act feel self-righteous. An individual also may devalue or dehumanize the recipient of the unethical act.

Through the use of these disengagement mechanisms, MDT suggests that individuals, who view themselves as ethical, deactivate the self-regulatory processes that would normally inhibit unethical behavior in order to validate or rationalize engaging in unethical behavior (Detert et al., 2008). Moreover, from a COR perspective, these disengagement mechanisms effectively serve to minimize the anticipated costs of unethical behavior, thereby providing a more favorable cost-benefit calculus in support of the unethical act. As a result, we offer the following:

*Hypothesis 1: When asked why individuals engaged in an unethical act they will report using a moral disengagement mechanism*.

### Ethical behavior and negative emotions: shame and guilt

Extant research predicts that individuals will compare their self-concept with “self-guides” (Higgins et al., 1985). Failure to match the self-concept with the personally relevant self-guide results in negative discrepancy-induced emotions (Strauman, 1996; Tangney et al., 1998). Higgins (1987) discusses these two broad categories of emotional syndromes as dejection-related and agitation-related emotions. Self-discrepancies that involve differences between an individual's actual attributes and what he/she wishes to attain or what others wish for him/her to attain are likely to result in dejection-related emotions, such as shame, whereas those that involve differences between an individual's actual attributes and what the individual feels he/she should attain or what others feel he/she should attain are likely to result in agitation-related emotions, such as feelings of guilt (Higgins, 1987; For a thorough discussion including examples that offer clarification on the difference between attributes an individual wishes vs. should attain please refer to Tangney and Dearing, 2002, p. 70–71). The greater the self-regulatory significance of the discrepancy, the greater level of emotional discomfort an individual will experience (Higgins, 1999; Bizman et al., 2001).

We view shame and guilt as emotional reactions to a perceived threat or loss of resources. Tangney (1991) and colleagues (Tangney et al., 1996) define guilt and shame as separate and distinct emotions that may promote or inhibit different behaviors (Tangney et al., 2005, 2007). Guilt refers to remorse over concerns that one's actions have potentially or actually caused harm, especially to others (Tangney et al., 1996; Ferguson and Stegge, 1998). In contrast to guilt, a person experiencing shame “focuses more on devaluing or condemning the entire self, experiences the self as fundamentally flawed, feels self-conscious about the visibility of one's actions, fears scorn, and thus avoids or hides from others” (Ferguson and Stegge, 1998, p. 20). Both guilt and shame cause personal distress resulting from perceived violations of one's moral standards (Tangney, 1991). However, they differ in focus; guilt leads to an external focus on the effects of one's behavior, whereas shame involves preoccupation on one's personal failings, deficiencies, and social standing. Guilty parties are more likely to take actions to protect or restore a relationship damaged by immoral or self-serving behavior. Comparatively, ashamed parties are more likely to take actions to defend or rebuild their own face or reputation. Thus, we propose:

*Hypothesis 2: Engaging in an unethical act results in negative emotions*.

### Beyond the unethical act: post-moral disengagement

Although previous research demonstrates the existence of negative emotions resulting from unethical behavior, the literature has been silent on the emotional reactions stemming from the realization of said behaviors. We argue that, despite coping with initial bouts of negative emotionality, as individuals learn of the consequences of their unethical behavior, their level of negative emotionality will intensify. Moreover, we hypothesize that the level of severity of the consequences will significantly influence the level of negative emotions such that the more severe the consequences (i.e., pain and suffering caused to others; public embarrassment; ostracized by friends) the greater the negative emotions. As we note above, shame is associated with preoccupation with how one's decisions affect the offender's self-esteem, reputation, or standing with others (Tangney, 1991; Tangney et al., 1996; Ferguson and Stegge, 1998). In contrast, guilt tends to be other-focused and involves attention to the adverse impact of one's actions on others as well as the relationship that connects them (Tangney, 1991; Tangney et al., 1996; Ferguson and Stegge, 1998). Regret for one's actions often motivates guilty parties to desire to make amends for their perceived wrongdoing (e.g., Ferguson and Stegge, 1998). Ashamed parties may ruminate on ways to repair their public image and regain lost social standing. This increased attention on self- and other- preservation is emotionally taxing and places the offender at risk of emotional exhaustion and burnout.

To cope with this post-decision dissonance (Festinger, 1957) and behavioral remorse (Oshikawa, 1969), we posit that individuals will engage in successive rounds of moral disengagement (i.e., post-moral disengagement). This implies a cycle in which (a) moral disengagement justifies the commission of an unethical act; (b) the unethical act creates negative emotional reactions; (c) realization of the consequences of the unethical act intensifies the underlying negative emotions; (d) post-moral disengagement is employed to lessen, interrupt or reverse the downward emotional spiral. Thus, we suggest the following:

*Hypothesis 3: Individuals will engage in post-moral disengagement in order to manage the negative feelings arising from engaging in an unethical act*.

*Hypothesis 4: There is a positive relationship between the severity of the outcome of the unethical act and the level of negative emotions experienced*.

*Hypothesis 5: Individuals will engage in post-moral disengagement in order to manage the negative emotions arising from the consequences of the unethical act*.

## Methods

### Sample

Participants were 191 students enrolled in an undergraduate business course at one of four universities in the U.S. The distribution of participants across the classes was 70, 54, 33, and 34 and all received extra credit for participating. Results from a MANOVA (Wilks' Lambda *F* = 0.558, *p* = 0.945, η^2^ = 0.02) with course as the independent variable and the variables of interest serving as the dependent variables revealed no significant differences. Thus, the four subgroups were combined to create one sample upon which all of our analyses were conducted. The sample consisted of 92 males (48%). Sixty percent (115) of the sample was Caucasian, 16 percent (30) was African-American, 24 percent (46) selected “other,” and the average age was 23.91 years.

### Procedure

Instructors of the courses forwarded an introductory email to their students that included a link to the electronic survey. Those who elected to participate in the study were randomly assigned to one of four conditions. All participants were asked to complete the pre-manipulation scales that included the moral person scale, scales that measured propensity to feel guilt and shame, and a moral disengagement scale. Next, participants were asked to read a scenario in which their friend “Pat” called them in the middle of the night, clearly inebriated, and asked for a ride home (see Box 1 for exact wording). After reading the scenario, participants completed the guilt and shame scales and in their own words described why they acted in the manner described in the scenario. Participants' descriptions were used to ensure that they accepted their role in the manipulation and to verify that they used a moral disengagement mechanism. Next, participants read a second scenario (Box 1) that revealed what happened to Pat after their phone call ended. Finally, respondents completed the guilt and shame measures again and scales measuring post-moral disengagement.

Box 1 **Manipulations.**

**DISCREPANCY MANIPULATION**

It is 11:00 p.m. on a Thursday night. You have been at the library since 5:00 p.m. studying for the final exam that you have tomorrow and decide to head back to your place to get a good night's sleep. Your friends left for an off-campus house party earlier in the evening and you told them that you would try to meet them later. They jokingly appointed you the designated driver even though you don't have a car. But they knew you had an important final exam that you needed to do well on, so they'll understand if you do not show. You get back to your place, get settled in, and start to doze off. After a while, the phone rings. You wake up and answer it. It's your friend Pat who sounds really drunk. Pat asks you to come drive them home. You and Pat have been friends for years now and you've never heard Pat sound this drunk. Pat's voice worries you, but since you don't have a car, you would have to walk over to the house party (about a mile away), drive Pat's car home, and then walk back to your place. You think to yourself “I really have to do well on this exam tomorrow and need my sleep. It's only a mile away…

**Discrepancy Condition**

…They can make it home safely.” You tell Pat that you really need to get your rest and that the drive isn't that far. Pat begs you to come saying “I drank way too much; I don't even remember where my car is.” You tell Pat to go find the car and drive slowly. Everything will be OK. You turn off your phone and fall back asleep.

**Control Condition**

…But they can't make it home safely.” You tell Pat that although you really need your rest you are on your way. Pat says “thank you, I drank way too much; I don't even remember where my car is.” You walk over to the house party, find Pat and the others, get them all in Pat's car and drive them back to Pat's place. Once at Pat's place, you stay until everyone has fallen asleep and then walk back to your place. You turn off your phone and fall back asleep.

**CONSEQUENCE MANIPULATION**

**Control Condition**

The next morning when you turn on your phone you see that you have a message from Pat. Pat is very thankful that you came to pick them up. Pat says that if it weren't for you, they wouldn't have made it home safely last night. Pat says that it is good to have friends like you who take their responsibility as a designated driver seriously.

**DUI Condition**

The next morning when you turn on your phone you see that you have a message from one of your friends. Pat was pulled over minutes after leaving the party. Pat was arrested and has been charged with a DUI (driving under the influence). Pat's license has been revoked and will not be allowed to drive for a year. No one was hurt. As the significance of this situation begins to sink in, you remember that you told Pat that everything would be OK.

**Single-Car Accident Condition**

The next morning when you turn on your phone you see that you have a message from one of your friends. Pat has been in an accident. Pat drove head on into a telephone pole minutes after leaving the party and has been taken to the hospital. The doctor thinks Pat will be able to go home tomorrow. Pat has been charged with a DUI (driving under the influence). Pat's license has been revoked and will not be allowed to drive for a year. No one other than Pat was hurt. As the significance of this situation begins to sink in, you remember that you told Pat that everything would be OK.

**Multi-Car Accident Condition**

The next morning when you turn on your phone you see that you have a message from one of your friends. Pat has been in a serious accident. Pat drove head-on into a minivan with a family of four in it minutes after leaving the party. Everyone was taken to the hospital with life-threatening injuries. The doctors do not think that the baby girl that was in the minivan is going to survive her injuries. Pat has been charged with a DUI (driving under the influence). Pat's license has been revoked and will not be allowed to drive for a year. As the significance of this situation begins to sink in, you remember that you told Pat that everything would be OK.

### Manipulations overview

Engagement in an unethical act was manipulated through a scenario (see Box 1). Individuals in the control condition learned that after Pat's phone call they picked up their friend and got Pat safely home. In the other three conditions, the participants learn that they remained in bed and told Pat to drive home carefully. Severity of the consequences of acting unethically was manipulated through a second scenario that explained the outcomes for Pat (see Box 1). There were four possible endings designed to successively increase the severity of the consequences for Pat. In the control condition (*N* = 46), Pat got home safely. In the DUI condition (*N* = 45), Pat received a DIU, lost his/her license, and spent the night in jail. In the single car accident condition (*N* = 48), Pat was in a single car accident, received a DIU, lost his/her license, and spent the night in the hospital. Finally, in the multi-car accident condition (*N* = 43), Pat was in a multi-car accident in which others were seriously injured, received a DIU, lost his/her license, and spent the night in the hospital.

### Manipulation generation and validation

To generate a manipulation that potential respondents would find relevant and unethical, we conducted a focus group with 50 business undergraduates attending a university in the U.S., which was not one from which we collected our data. Participants in the focus group included 16 men (32%), 34 (68%) were Caucasians, and ranged in age from 20 to 24. We began the session by describing a scenario in which an individual cheating on an exam. Discussions clearly indicated that the participants did not view this act as unethical. So, we opened the floor to the group and asked them to offer examples of unethical situations they often face. Situations offered included cheating in a relationship, shoplifting, and lying to one's parents. However, the ethicality of these situations was not unanimous. One situation that did unite the participants was allowing a friend to drive drunk. Based on their feedback we developed a manipulation in which respondents were told they allowed a friend to drive drunk.

To ensure our manipulation was valid, we needed to confirm that allowing a friend to drive drunk was a behavior in which potential respondents had engaged. To test this assertion, we asked 129 students—66 (51%) male, 98 (76%) classified as Juniors or Seniors, and 108 (84%) Caucasian—enrolled in business classes at a university in the U.S., which was not one from which our data were gathered, to complete a 12-item survey. Items on the survey asked them to indicate whether they had engaged in the behaviors listed including whether they allowed a friend to drive drunk and whether they had ridden in a car with a driver who was under the influence. A total of 101 (78%) answered yes to one or both of these statements. These results suggest that the behavior assigned to our respondents in the manipulation scenario was one that was common to respondents from our population of interest.

We conducted a pretest to confirm that our scenarios were viewed as plausible and relevant. The pretest sample was composed of 40 undergraduate students enrolled in a business course at a university in the U.S., which was not one from which our data were gathered. The average age of the respondents was 22.05 years, 22 (55%) were male, and 31 (78%) were Caucasian.

To ascertain whether the respondents accepted the actions described in the scenario as their own, we content analyzed the responses to the open-ended question: Why did you let Pat drive drunk? The vast majority of the respondents (90%) offered a reason that aligned with the initial scenario (e.g., “Because I had already told him I had a huge test tomorrow and needed rest”). Only 4 (10%) indicated that they would not have acted as the scenario suggested (e.g., “I wouldn't have told Pat to drive. I would have told him to spend the night there”).

To test whether our outcome manipulation was successful, we conducted a MANOVA with the outcome condition serving as the independent variable and each of the manipulation check items serving as the dependent variables. The overall omnibus *F*-test was significant (Wilks' Lambda *F* = 88.74, *p* < 0.001, η^2^ = 0.63). Results for the follow up one-way ANOVAs using a Scheffe *post-hoc* test, showed that three of our manipulation check items were significant and the means were as expected. However, one of the manipulation check items (“Although Pat was in an accident, he did not go to the hospital.”) failed to distinguish differences among the groups. Upon further review, we determined that the double-barreled nature of the item confused the respondents. To correct this problem, we changed the wording of the item to “Pat was in a single car accident.”

Finally, we analyzed the results for the presence of demand characteristics. In order to determine if the respondents were able to ascertain the true purpose of our study we asked them to tell us what they thought the survey was about by selecting one of three options: How individuals respond to life situations (our cover story), I don't know, or other followed by blank to indicate in their own words the purpose of the study. Results from an ANOVA (*F* = < 1, *p* = 0.56, η^2^ = 0.05) revealed no differences in the responses to the demand characteristic check item across the conditions. The majority (29 or 73%) selected our cover story explanation, an additional 4 (10%) indicated that they did not know, and the remaining 7 (18%) selected other. None of the open-ended comments (e.g., self-esteem, self-values) mentioned the true purpose of the study. With the exception of the one manipulation check item, which we changed for use in the experiment, these results demonstrated that the manipulations were effective.

### Initial manipulation confirmation

Prior to conducting any analyses, we first confirmed that the respondents in our experimental conditions accepted their role in the manipulation. Just as we did in the pretest analyses, we asked the respondents to tell us in their own words why they let Pat drive drunk. This open-ended question appeared after the time 1 guilt and shame scales for those not in the control group. We removed the respondents (9 or 7%) who indicated that they would not have acted this way (e.g., “I wouldn't have let him drive drunk.”) from the sample dropping our overall *N* to 182.

### Outcome manipulation confirmation

We conducted a MANOVA to test whether our outcome manipulation was successful. Specifically, the outcome condition served as the independent variable and each of the manipulation check items served as the dependent variables. The overall omnibus F-test was significant (Wilks' Lambda *F* = 70.32, *p* < 0.001, η^2^ = 0.588). Results for the follow up one-way ANOVAs using a Scheffe *post-hoc* test, shown in Table 1, reveal that each of our manipulation check items was significant and the means were as expected confirming that outcome manipulation was successful.

**Table 1:** Manipulation check results.

			**Control (*N* = 46)**	**DUI (*N* = 45)**	**Single-car accident (*N* = 48)**	**Multi-car accident (*N* = 43)**
**Manipulation check item**	***F***	***p***	***X***	***X***	***X***	***X***
I made sure Pat did not drive drunk by fulfilling my role as the designated driver.	78.74	0.00	4.33^a^	1.67^b^	1.46^b^	1.63^b^
Pat received a DUI.	86.20	0.00	1.61^a^	4.51^b^	4.58^b^	4.30^b^
Pat was in a single car accident.	73.08	0.00	1.48^a^	1.73^a^	4.42^b^	1.81^a^
Pat was in an accident that involved another vehicle.	84.53	0.00	1.50^a^	1.53^a^	1.50^a^	4.37^b^

*N = 182. df = 3,178. Means in the same row with the same subscript are not significantly different from one another*.

### Pre-manipulation measures

#### Moral person

Prior to being exposed to the initial manipulation, we measured the degree to which the participants viewed themselves as moral individuals with a 15-item scale (α = 0.75) developed by Sekerka et al. (2009). Reponses were made on a 5-point Likert scale (1 = Definitely not true of me and 5 = Definitely true of me). A sample item is “I am determined to do the right thing.”

#### Propensity to feel guilt and shame

Prior to being exposed to the initial manipulation, we administered 11-items from Tangney et al. (2000) guilt and shame scale (TOSCA-3) to determine whether the participants had the ability to feel guilt and shame (α = 0.62). The items in the scale describe potential situations (e.g., you broke something at work) while the responses describe both a guilt (e.g., You would think “This is making me anxious. I need to either fix it or get someone else to.”) and a shame (e.g., You would think about quitting.) reaction to the situation. Participants indicated how likely they were to act in the manner described by both the guilt and the shame reactions on a 5-point Likert scale (1 = Very unlikely and 5 = Very likely) of the scale.

#### Moral disengagement

Prior to reading the initial manipulation, participants completed the 32-item (α = 0.93) moral disengagement scale (Bandura et al., 1996). Reponses were made on a 7-point Likert scale (1 = Strongly disagree and 7 = Strongly agree). A sample item is “People cannot be blamed for misbehaving if their friends pressured them to do it.”

### Post-manipulation measures

#### Guilt

We used the 6-item guilt scale developed by Kubany et al. (1996) to measure guilt at two points in time: after the initial scenario (α = 0.93) and after the final scenario (α = 0.94). A sample item is “I did something that I should not have done.” Responses were made on a 5-point Likert scale (1 = Not at all true and 5 = Extremely true).

#### Shame

We used the 4-item shame scale developed by Andrews et al. (2002) to measure shame at two points in time: after the initial scenario (α = 0.73) and after the final scenario (α = 0.79). A sample item is “How likely are you to try and cover up or conceal what you did from your friends?” Responses were made on a 5-point Likert scale (5 = Very likely and 1 = Very unlikely).

#### Post-moral disengagement

To measure post-moral disengagement, we created 11 statements that mapped to Bandura's (1986) four moral disengagement categories. The items are shown in Table 2. To explore the underlying factor structure of these items, we conducted an exploratory factor analysis with SPSS 18 using a principal axis factoring extraction method and an oblimin rotation. The data used for these analyses (*N* = 136) came from the individuals in the three manipulated outcome conditions because those in the control group did not respond to these items. Applying a factor-loading cut off criteria of 0.45 produced two factors with no cross-loadings (see Table 2) and one item that we dropped because it failed to load on either factor. The first factor, which we named diffusing and displacing responsibility, contained all of the items we developed to map to Bandura's (1999) second and fourth moral disengagement categories. These categories include disengagement practices that minimize personal involvement and blame or devalue the recipients. The second factor, which we named minimizing and reconstruing actions, includes all of the items we created to map to Bandura's (1999) first and third moral disengagement categories. These categories include reconstruing one's conduct so that it is not considered immoral and misrepresenting or discounting the resulting negative consequences. We created one scale for each factor by averaging the items that loaded on the factor. The resulting Cronbach alphas were 0.87 and 0.77 respectively.

**Table 2:** Factor loadings for 11-item post-moral disengagement measure.

**Item**	**Diffusing or displacing responsibility**	**Minimizing and reconstruing actions**
Make it clear to Pat that you recognize that your action of not providing a ride was a grave misstep, but that you felt you had no other alternatives.	**0.89**	
Tell Pat that there were circumstances beyond your control, which caused you to wrongly refuse to give Pat a ride.	**0.86**	−0.11
Tell Pat that you recognize that not picking Pat up was a terrible thing to do, but remind Pat that Pat's timing gave you no choice.	**0.75**	
Explain to Pat that while your actions were bad, you did the best you could at the time	**0.63**	
Make sure Pat knows that not giving Pat a ride was a critical error, but then explain to Pat why you could not have prevented the outcome of the evening.	**0.49**	0.29
Take responsibility for not giving Pat a ride, but then point out that your actions could have been worse.		**0.77**
Let Pat know that you are responsible for not providing a ride, but that this act alone is not bad.		**0.68**
Admit that you did not give Pat a ride, but remind Pat that your actions did not hurt anyone.		**0.63**
Make sure Pat knows that you understand that you are responsible for not giving Pat a ride, but explain how the situation may turn out to be a benefit Pat in the long-run.		**0.58**
Accept responsibility for not giving Pat a ride, but try to make your actions appear less severe than they actually are.	0.16	**0.47**
Let Pat know that while not providing a ride was a serious slip, others' behaviors contributed to the evening's events too.	0.35	0.38
Eigenvalue	4.38	1.03
Variance explained	39.79	9.40

*Loadings > 0.45 are bolded; loadings < 0.10 are suppressed*.

#### Manipulation check items

At the end of the survey, participants were asked to complete a 4-item manipulation check scale. The four items included “I made sure Pat did not drive drunk by fulfilling my role as the designated driver.” (control group), “Pat received a DUI.” (all groups except the control group), “Pat was in a single car accident.” (single-car accident condition), and “Pat was in an accident that involved another vehicle.” (multi-car accident condition). Responses were made on a 5-point Likert scale (1 = Strongly disagree and 5 = Strongly agree).

## Results

### Descriptive statistics

Table 3 shows the means, standard deviations, and correlations among the measured variables of interest. As the correlations between our post-moral disengagement scales exceeded 0.50, we followed the procedure outlined by Fornell and Larcker (1981) to test the discriminant validity of our scales. Specifically, we calculated the square root of the average variance explained for all variables. To demonstrate discriminant validity, this value (presented on the diagonal in Table 3) must exceed the corresponding latent variable correlations in the same row and column. If this condition is met, then there is evidence that the variance shared between any two constructs (i.e., the correlation) is less than the average variance explained by the items that compose the scale (i.e., square root of the average variance). As shown in Table 3, this condition is met for the six unique variables in our study. This condition is not met, nor was it expected to be, for the measures that used the same items (i.e., propensity to feel guilt and shame) or that were measured at two points in time (i.e., guilt and shame).

**Table 3:** Correlations, means, and standard deviations.

**Variable**	**Mean**	**SD**	**1**	**2**	**3**	**4**	**5**	**6**	**7**	**8**	**9**	**10**
1. Moral person^a^	3.90	0.44	**0.56**									
2. Propensity to feel guilt^a^	3.54	0.34	0.17^*^	**0.38**								
3. Propensity to feel shame^a^	3.25	0.49	0.09	0.37^***^	**0.33**							
4. Moral disengagement^a^	2.53	0.85	−0.29^***^	−0.15^*^	−0.06	**0.58**						
5. Guilt time 1^a^	2.72	0.60	0.08	0.12	0.23^**^	−0.11	**0.75**					
6. Shame time 1^a^	3.02	0.96	0.03	0.14	0.34^***^	−0.04	0.48^***^	**0.62**				
7. Guilt time 2^a^	2.83	0.65	0.06	0.15^*^	0.23^**^	−0.02	0.78^***^	0.49^***^	**0.73**			
8. Shame time 2^a^	3.14	1.08	−0.01	0.19^*^	0.29^***^	0.06	0.41^***^	0.80^***^	0.50^***^	**0.68**		
9. Diffusing and displacing responsibilities^b^	2.76	1.00	−0.04	0.09	0.16	0.26^**^	−0.25^**^	0.12	−0.25^**^	0.24^**^	**0.76**	
10. Minimizing and reconstruing actions^b^	2.39	0.86	−0.11	0.08	0.10	0.39^***^	−0.10	0.19^*^	−0.07	0.27^**^	0.51^***^	**0.64**

a *N = 182*.

b *N = 136*.

* p < 0.05;

** p < 0.01;

*** *p < 0.001. Values on the diagonal are the square root of the average variance explained which must be larger than all zero-order correlations in the row and column in which they appear to demonstrate discriminant validity (Fornell and Larcker, 1981)*.

### Pre-manipulation scale results

Prior to reading the initial scenario, participants completed the moral person, moral disengagement, and propensity to feel guilt and shame scales. These scales were used as baseline indicators for the degree to which the respondents viewed themselves as moral individuals who were capable of morally disengaging and feeling guilt and shame. This step was essential to our being able to create a discrepancy. To ensure that the participants did not differ significantly on these measures, we conducted a MANOVA with the manipulated condition as the independent variable and the four pre-manipulation scales as the dependent variables. The overall omnibus *F*-test (Wilks' Lambda *F* = < 1, *p* = 0.783, η^2^ = 0.02) was not significant. The means for moral person ranged from 3.85 to 3.99, from 2.44 to 2.67 for moral disengagement, from 3.48 to 3.57 for propensity to feel guilt, and from 3.19 to 3.31 for propensity to feel shame. These results demonstrate no significant differences between the individuals on the four pre-manipulation variables. Thus, the randomization process was successful and any mean differences among the groups can be attributed to the manipulations.

### Moral disengagement

To test Hypothesis 1, we needed to demonstrate that individuals, who engaged in an unethical act, reported using a moral disengagement mechanism. To do this we examined the responses to the open-ended question: Why did you let Pat drive drunk? Specifically, two of the authors independently coded the responses (*N* = 136) from the participants assigned to the three manipulated conditions into as many of Bandura's (1986) four categories of moral disengagement as appropriate. For instance, the statement “Because I had an important exam that I needed rest for” was coded into the first category because the individual reframed the act as personally acceptable while the statement “I was concerned of my test in the morning and justified him driving because he was only driving a short distance” was coded into the first category for the same reason and the third category because the individual also discounted the negativity of telling Pat to drive drunk. The agreement rate was 96% (131/136). Another author served as the tiebreaker and coded the 5 statements where agreement was not reached. In each of these cases, the additional author matched one of the original codes.

The majority (89 or 61%) of the reasons for letting Pat drive drunk were coded into the first category. To fall into this category, respondents needed to reconstrue their conduct so that it was not considered immoral. To accomplish this, they described their behavior as socially and personally acceptable. Some sample items include “Because I had to get some rest for the test,” or “Because I had a final exam in the morning.” The second category involved minimizing one's involvement. A total of 26 (18%) responses were placed in this category. Sample items include “There was no agreement that I would be the designated driver,” or “I don't have a car to drive them in the first place.” Only 10 (7%) of the responses were coded into the third category that required respondents to misrepresent or discount the negative consequences. Sample items in this category include “He was only driving a short distance,” or “It was only a mile away, if he drove slowly, it would not be as dangerous.” Finally, 22 (15%) of the responses were placed into the fourth category. Responses in this category blamed or dehumanized Pat, the recipient of the unethical act. Sample items include “He needs to be responsible for his own actions,” or “Pat shouldn't have gotten that drunk.” These results offer support for Hypothesis 1 as the reasons given for acting in an unethical manner fell into Bandura's (1986) moral disengagement categories.

### Negative emotions

To test Hypothesis 2, we needed to establish that individuals who acted ethically (i.e., control group) felt less negative emotions than those who engaged in an unethical act (i.e., the manipulation groups). To do this, we compared the time 1 guilt and shame scores for the control group, who did the right thing, to those in the manipulation conditions, who remained in bed. Individuals in the control condition should not feel negative emotions because they did what a “moral person” would do. Those in the manipulated conditions should feel negative emotions because, although they view themselves as a moral person based on the pre-manipulation results, they failed to do what a “moral person” should do.

Results from a MANOVA in which guilt and shame at time 1 served as the dependent variables and the experimental condition served as the independent variable revealed a significant overall omnibus *F*-test (Wilks' Lambda *F* = 10.45, *p* < 0.001, η^2^ = 0.15). The between-subjects effects demonstrated a main effect for each guilt (*F* = 15.50, *p* < 0.001, η^2^ = 0.21) and shame (*F* = 15.02, *p* < 0.001, η^2^ = 0.20). A Scheffe *post-hoc* test showed that the means for guilt (2.26) and shame (2.28) for the control group were significantly lower than the means from the other three conditions (2.81 and 3.25 DUI; 2.89 and 3.25 single-car accident; 2.93 and 3.33 multi-car accident, respectively; *p* < 0.001) and that the means in the other three conditions were not significantly different from one another, as displayed in Table 4. Thus, Hypothesis 2 was supported as we can conclude that the individuals in the manipulated conditions felt negative emotions after engaging in an unethical act compared to those in the control condition.

**Table 4:** Mean comparisons by condition for guilt and shame at time 1 and time 2.

**Condition**	***N***	**Guilt**				**Shame**			
		**Time 1**	**Time 2**	***t***	***p***	**Time 1**	**Time 2**	***t***	***p***
Control	46	2.26	2.29	−0.53	0.60	2.28	2.27	0.08	0.94
DUI	45	2.81	2.85	−0.63	0.53	3.25	3.34	−1.21	0.23
Single-car	48	2.89	3.10	−4.53	0.00	3.24	3.44	−1.71	0.08
Multi-car	43	2.93	3.09	−1.97	0.05	3.33	3.50	−2.31	0.02

*N = 182*.

### Managing the negative feelings from the unethical act

Given that Hypothesis 2 was supported, we next investigated whether individuals will engage in post-moral disengagement in order to manage the negative emotions felt. To test this assumption, we correlated the post-moral disengagement scales with guilt and shame at time 1 as these values reflect the subjects' awareness of their engagement in an unethical act. Two of the four correlations, shown in Table 3, were significant. Minimizing and reconstruing actions was significantly correlated with shame at time 1 indicating that as the level of shame one feels increases so does the use of this form of post-moral disengagement. While the correlation between guilt at time 1 and diffusing and displacing responsibilities was significant, it was negative, which is opposite of what we predicted.

We also regressed each factor of moral disengagement on guilt and shame measured at time 1 in order to take into account the variance explained by each after accounting for the other. These analyses are displayed on Table 5. Multi-collinearity was assessed via two commonly used indices: tolerance and variance inflation factor (VIF), where tolerance values should be close to one and VIF scores should be less than two (Miles and Shelvin, 2003). There is no evidence of collinearity in our results. We find a positive and significant relationship between shame and both diffusing and displacing responsibilities and minimizing and reconstruing actions (*B* = 0.321, *p* = 0.004 and *B* = 0.286, *p* = 0.004, respectively). We also find that there is a negative and significant relationship between guilt and each factor of the moral disengagement scale (*B* = −0.606, *p* < 0.001 and *B* = −0.304, *p* = 0.028, respectively). Guilt and shame explain 11.5% of the variance in diffusing and displacing responsibilities and 6.9% of the variance in minimizing and reconstruing actions. Hypothesis 3 received partial support; while both guilt and shame predicted moral disengagement, guilt was in the opposite direction that we were expecting.

**Table 5:** Regression results for the effects of negative emotions (guilt and shame) on moral disengagement.

	**Time 1**		**Time 2**	
	**Diffusing and displacing responsibilities**	**Minimizing and reconstruing actions**	**Diffusing and displacing responsibilities**	**Minimizing and reconstruing actions**
Intercept	3.464 (0.457)^***^	2.326 (0.403)^***^	3.194 (0.424)^***^	2.127 (0.389)^***^
Guilt	−0.606 (0.155)^***^	−0.304 (0.137)^*^	−0.603 (0.137)^***^	−0.283 (0.122)^*^
Shame	0.321 (0.111)^**^	0.286 (0.098)^**^	0.407 (0.092)^***^	0.325 (0.082)^***^
	***0.842/1.188***	***0.842/1.188***	***0.865/1.157***	***0.865/1.157***
*F*	8.679	4.953	14.275	8.205
*R*^2^	0.115	0.069	0.117	0.110
Adj*R*^2^	0.102	0.055	0.164	0.096
*p*-value	<0.001	0.008	<0.001	<0.001

*N = 136. df = 2,133 for each model. Standard error for each estimate in parentheses. All beta coefficients are unstandardized. Tolerance and VIF are in italic bold respectively*.

* p < 0.05;

** p < 0.01;

*** *p < 0.001*.

### Severity of the outcome

The outcome scenarios were designed to successively increase the consequences for Pat. Hypothesis 4 predicted that the levels of guilt and shame would be positively related to the negative consequences of the unethical act. To test this contention, we conducted a MANOVA with condition serving as the independent variable and guilt and shame at time 2 as the dependent variables, as displayed in Table 4. The overall omnibus F-test was significant (Wilks' Lambda *F* = 12.81, *p* < 0.001, η^2^ = 0.18). The between-subjects effects demonstrated a main effect for guilt (*F* = 21.05, *p* < 0.001, η^2^ = 0.26) and shame (*F* = 16.51, *p* < 0.001, η^2^ = 0.22). The Scheffe *post-hoc* revealed that the control group had the lowest level of guilt (2.29) and shame (2.27) and that these means were significantly lower than the means of the three other conditions. However, the means for guilt and shame in the other three conditions (2.85 and 3.34 DUI, 3.10 and 3.44 single-car accident, and 3.09 and 3.50 multi-car accident, respectively) were not significantly different from one another. Thus, while knowing the consequences for Pat did increase respondents' levels of guilt and shame, the severity of the outcomes did not differentially increase these feelings. Thus, no support was found for Hypothesis 4.

### Post-moral disengagement

Our previously reported findings demonstrate that individuals do feel negative emotions after learning the consequences of their unethical act. Our final hypothesis proposes a way in which individuals may deal with these feelings. Specifically, we predicted that individuals will engage in post-moral disengagement in order to manage the negative emotions felt. To test this assumption, we correlated the post-moral disengagement scales with guilt and shame at time 2 as these values reflect the subjects' awareness of the consequences for Pat. Three of the four correlations, shown in Table 3, were significant. Both correlations with shame at time 2 were significant indicating that as the level of shame one feels increases so does the use of both forms of post-moral disengagement. However, only the correlation between guilt at time 2 and diffusing and displacing responsibilities was significant, and this correlation was negative.

We regressed each factor of moral disengagement on guilt and shame measured at time 2. These analyses are displayed on Table 5. Multi-collinearity was not an issue with these data. We find a positive and significant relationship between shame and both diffusing and displacing responsibilities and minimizing and reconstruing actions (*B* = 0.407, *p* < 0.001 and *B* = 0.325, *p* < 0.001, respectively). We find that there is a negative and significant relationship between guilt and each factor of the moral disengagement scale (*B* = −0.603, *p* < 0.001 and *B* = −0.283, *p* = 0.022, respectively). Guilt and shame explain 11.7% of the variance in diffusing and displacing responsibilities and 11.0% of the variance in minimizing and reconstruing actions. Combined, these results partially support Hypothesis 5. Hypothesis 5 was supported for shame, but not for guilt, as the relationship between guilt and moral disengagement was found to be negative instead of positive, which is opposite of what we predicted.

### *Post-hoc* tests

**Figure 2:**
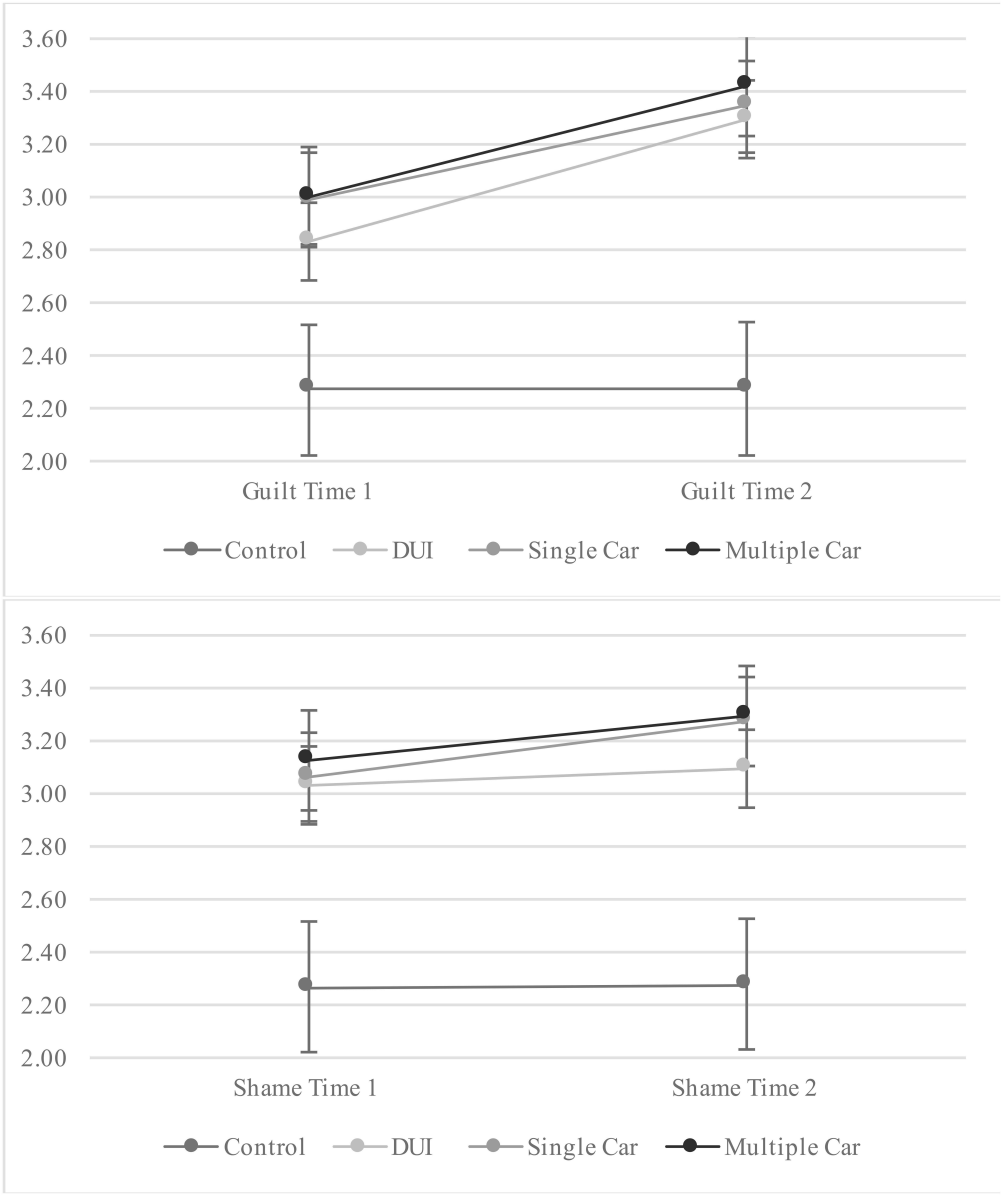
Comparisons of time 1 and time 2 by condition for guilt and shame.

We were also interested in whether participants would experience a change each in guilt and shame between the time they engaged in the unethical act (time 1) and the time they were told of the consequences of their act (time 2). We used a repeated measures MANOVA where condition served as the between-subjects factor, time (1 and 2) was the within-subjects factor and guilt and shame served as the dependent variables. The omnibus *F*-test suggests that there was an effect of time [Wilks' Lambda *F*_(2, 177)_ = 7.84, *p* = 0.001, η^2^ = 0.08], but no difference between the conditions by time [Wilks' Lambda *F*_(6, 354)_ = 1.38, *p* = 0.22, η^2^ = 0.04] on guilt and shame. Univariate tests also indicated that there was not a condition effect on either guilt [*F*_(3, 178)_ = 2.27, *p* = 0.082, η^2^ = 0.02] or shame [*F*_(3, 178)_ = 0.924, *p* = 0.43, η^2^ = 0.02] across time, but there was a time effect for each guilt [*F*_(1, 178)_ = 12.93, *p* < 0.001, η^2^ = 0.07] and shame [*F*_(1, 178)_ = 5.47, *p* = 0.02, η^2^ = 0.03], as displayed visually in Figure 2. Interestingly, but not surprisingly, we find that learning of the consequence of one's act produces increased levels of negative emotions compared to simply knowing that one has engaged in an unethical act. However, these effects did not differ by condition.

## Discussion

### Contributions and implications for the behavioral ethics literature

Our study contributes to the field of behavioral ethics in several ways. First, we offer a potential explanation of how individuals, who view themselves as moral, are able to both engage in and subsequently cope with an unethical act. To navigate these decisions, MDT posits that individuals are able to justify their unprincipled behavior and reduce feelings of condemnation or self-censure through moral disengagement. However, they still experience negative emotions as a result of the inconsistency between their behavior and their self-guide. Our results suggest that behavioral ethicists should continue to explore not only the causes of individual behavior but also the psychological consequences imposed on the individual after engaging in such acts.

Second, our study extends MDT by examining what happens after the negative emotions that result from having engaged in the unethical act. Research has not ventured far past exploring the immediate outcomes resulting from behaving unethically (Detert et al., 2008; Tillman et al., 2017), yet it indicates that individuals will experience a greater magnitude of negative emotions after having learned that one's behavior was responsible for causing a negative outcome (Kubany and Watson, 2003). Our study sought to explore the emotions that individuals experience after learning the consequences of their unethical behavior and how they cope with these emotions. Our results indicate that individuals did experience greater negative emotions after having learned the consequences of their unethical behavior compared to the emotions experienced immediately following the act. In addition, and in line with previous work (Johnson and Connelly, 2016), we find that guilt and shame relate differently to moral disengagement. Our findings suggest shame was positively related and guilt was negatively related to moral disengagement, implying that those who felt higher levels of guilt to some extent accepted fault for the outcome and did not rationalize it by morally disengaging. Experiencing a negative emotion does not universally lead to moral disengagement coping strategies; shame may drive individuals to morally disengage to save their reputation. Conversely, guilt may reduce moral disengagement as individuals seek actions to rectify the immoral action in other ways.

Third, our study contributed to the extension of MDT by examining whether the severity of the known consequences influences the level of negative emotions felt by individuals. We proposed that the more severe the consequence the greater the level of negative emotions individuals would experience. Our results indicate, however, that despite the various degrees of severity in the consequences of one's actions, individuals' level of negative emotions did not change significantly. These findings suggest that just learning of the negative consequences of one's unethical actions will cause individuals to experience negative emotions.

Finally, the present study examined whether moral disengagement serves as a multi-stage regulatory mechanism. Although moral disengagement has been shown to be a proactive regulatory mechanism that allows individuals to engage in unethical behavior, our study seems to suggest that it also may serve as a coping mechanism that allows individuals to deal with the negative emotions that arise after knowing the consequences of an unethical act. The implication here is there is a need for individuals who engage in unethical behaviors to continue to convince themselves and others that their behavior was justified. Thus, we encourage behavioral ethics researchers to continue exploring the nature of explanatory mechanisms at different stages in the ethical decision-making process.

### Implications for practice

Although our study's main focus was to test our theoretical hypotheses, the results of this study offer several important practical implications. Past research has shown that emotions that are the result of negative experiences can be functional (e.g., Haidt, 2003; De Hooge et al., 2008, 2011). Our findings suggest that individuals who felt higher levels of guilt after learning about the consequences of their unethical act did not attempt to rationalize their actions by morally disengaging. This finding may be considered functional if these individuals did not revert to morally disengaging as a means of coping with the negative emotions and rather assumed responsibility for the consequences of their unethical actions or resorted to other actions aimed at rectifying their immoral act. Our findings offer a preliminary glimpse into how individuals who experience feelings of guilt after learning of the consequences of their unethical act cope with the negative emotions, but further investigation aimed at examining the lingering effects of guilt as well as its influence on future ethical decision-making is warranted.

In contrast, our findings indicate that individuals who experienced higher levels of shame after learning about the consequences were more likely to morally disengage than their counterparts. Individuals who experience shame may morally disengage in an effort to save their reputation since shame involves a preoccupation with one's social standing. Typically shame is associated with feelings of anger and of inferiority (Allan et al., 1994), and individuals might cope by rationalizing or avoiding the situation altogether to reduce their discomfort, as this study would suggest. Alternatively, previous research suggests that under specific conditions, such as when the presence of shame is relevant to one's pursuit of goals, shamed individuals may be more likely to engage in prosocial behaviors to defend, rebuild, and regain their standing (De Hooge et al., 2008). While under these conditions, these individuals may engage in good behaviors toward those who may have been impacted by their acts to recuperate their social standing, but at what opportunity cost? As these individuals attempt to repair damaged relationships, others in the individuals' environment may be ignored.

### Limitations and future directions

Although this study makes a number of contributions to the extant literature, there are limitations. First, because our sample was composed of college students, caution should be used in generalizing the results from our study (Gordon et al., 1986). However, we feel our sample is appropriate given the focus on psychological processes and emotional consequences. Various researchers (e.g., Greenberg, 1987) have concluded that student samples do not present problems above and beyond other samples and may be appropriate and useful in studying psychological processes. We ensured that the scenario tested was applicable to the population studied. Nevertheless, future research should utilize more diverse samples in order to test the generalizability of the findings.

Second, our study design did not allow participants to decide whether or not to engage in an unethical act. Instead, the participants were provided with a discrepancy to evaluate. As a result, it is possible that individuals made their retrospective decisions about moral disengagement with this in mind. Although results of our pretests confirmed that the vast majority of the sample has engaged in the assigned act and view it as an unethical behavior, future research may benefit from an approach that incorporates extensive interviews about individuals' own personal experiences to more accurately assess the ethicality of the event, and the relevance to self-guides and interpersonal interactions. A retrospective look at such acts also will allow researchers to investigate the notion that moral disengagement is a continuous process that allows individuals to continue with the act even after consideration and initial engagement. Extensions of this work might also consider the complex interplay of unethical responses by multiple actors, and not only by the protagonist. Additionally, future research may explore the effects of continuous moral disengagement; that is, as individuals disengage in order to act unethically and subsequently rely on disengagement as a coping strategy, does the process of morally disengaging become easier to validate?

Finally, the current research tested only negative emotions. Future research should explore positive emotions such as happiness or satisfaction. Just as high levels of discrepancies produce feelings such of guilt, contempt, anger, or shame, low levels of discrepancy or the absence of the discrepancies in various self-guides should likewise produce more positive feelings.

## Conclusion

The present study integrated MDT and COR to provide a potential examination into the process by which individuals, who view themselves as moral, disengage to commit unethical acts and the negative emotions that result when these individuals learn the consequences of their unethical behavior. We suggest that individuals may turn to moral disengagement not only to validate engaging in an unethical act, but to re-validate their decision after learning the negative consequences of their behavior. Disengaging after learning the negative consequences of one's unethical act serves as a means of managing relationships with those who were hurt as a result of the act.

## Ethics statement

This study was carried out in accordance with the recommendations of “University of Alabama Institutional Review Board (IRB)” with written informed consent from all subjects. All subjects gave written informed consent in accordance with the Declaration of Helsinki. The protocol was approved by the “University of Alabama IRB.”

## Author contributions

All authors contributed substantively to the design, development, revisions, and intellectual property presented in this paper.

### Conflict of interest statement

The authors declare that the research was conducted in the absence of any commercial or financial relationships that could be construed as a potential conflict of interest.
